# Comparative Transcriptional Profiling of Two Wheat Genotypes, with Contrasting Levels of Minerals in Grains, Shows Expression Differences during Grain Filling

**DOI:** 10.1371/journal.pone.0111718

**Published:** 2014-11-03

**Authors:** Sudhir P. Singh, Raja Jeet, Jitendra Kumar, Vishnu Shukla, Rakesh Srivastava, Shrikant S. Mantri, Rakesh Tuli

**Affiliations:** 1 National Agri-Food Biotechnology Institute, Department of Biotechnology (DBT), C-127, Industrial Area, Phase VIII, Mohali, 160071, India; 2 School of Biotechnology, Banaras Hindu University, Varanasi, India; Institute for Sustainable Agriculture (IAS-CSIC), Spain

## Abstract

Wheat is one of the most important cereal crops in the world. To identify the candidate genes for mineral accumulation, it is important to examine differential transcriptome between wheat genotypes, with contrasting levels of minerals in grains. A transcriptional comparison of developing grains was carried out between two wheat genotypes- *Triticum aestivum* Cv. WL711 (low grain mineral), and *T. aestivum* L. IITR26 (high grain mineral), using Affymetrix GeneChip Wheat Genome Array. The study identified a total of 580 probe sets as differentially expressed (with *log2* fold change of ≥2 at p≤0.01) between the two genotypes, during grain filling. Transcripts with significant differences in induction or repression between the two genotypes included genes related to metal homeostasis, metal tolerance, lignin and flavonoid biosynthesis, amino acid and protein transport, vacuolar-sorting receptor, aquaporins, and stress responses. Meta-analysis revealed spatial and temporal signatures of a majority of the differentially regulated transcripts.

## Introduction

Micronutrients play an important role in metabolism, such as in the production and functioning of enzymes, hormones and other substances. Therefore, an adequate intake of trace elements is necessary for proper growth and development [1]. Prevalence of mineral deficiency, especially of iron and zinc, has been estimated in more than two billion people worldwide (http://www.unicef.org/). In developing countries, every second pregnant woman and about 40% of preschool children are estimated to be affected by the mineral micronutrient deficiency (http://www.who.int/nutrition/topics/ida/en/). The extent of reliance on staple crop based diet is one of the major reasons for mineral micronutrient malnutrition in human beings, mainly in resource-poor countries [2]. Development of mineral enriched cereal grains through breeding or biotechnological interventions is important for addressing micronutrient insufficiency [3].

Bread wheat (*Triticum aestivum* L.) is a globally important cereal crop, accounting for 20% of the world’s daily food supply [4]. Most high yielding wheat cultivars have low content of grain mineral nutrients [5], [6]. The primitive wheat genotypes constitute a pool of significant variability for grain micronutrients, which can be utilized for breeding and/or genetically engineering wheat cultivars [7]. Landraces, the primitive cultivated genotypes, often exhibit elevated grain mineral concentration and biotic and abiotic stress tolerance [8], [9]. For example, a primitive cultivar, *T. aestivum* L. IITR26 exhibits higher grain mineral concentration than the post-green revolution modern cultivar, *T. aestivum* Cv. WL711 [10], [11], [12]. We have earlier reported a distinct level of mineral distribution in maternal and filial grain tissues of the two wheat genotypes, IITR26 and WL711 [10], [11]. IITR26 is more efficient in accumulating micronutrients (e.g. Fe, Zn and Mn) in grain tissues than WL711. Furthermore, comparatively higher occurrence of nutritionally important minerals was observed in the endosperm of IITR26 grains, than WL711 [10], [11]. Differential expression profiling of genotypes with contrasting characters is an efficient tool for understanding the molecular basis of such phenotypic differences [13]. Therefore, comparative transcriptional profiling in the two wheat genotypes, IITR26 and WL711, during grain development was done in this study.

The three major grain developmental phases in wheat are 6–10 days after anthesis (DAA), 12–21 DAA and 28–42 DAA [14] (http://www.wheatbp.net). During grain filling, mineral nutrients are transported by the maternal tissues to filial grain tissues via endosperm cavity and transfer cells [15], [16]. Grain growth rate has been recorded as the highest during 7^th^ to 28^th^ DAA and lowest during 28 DAA till maturity [17]. The grain developmental stages at, 14 and 28 DAA are major transition phases in wheat grain development with distinctive pattern of transcript abundance [14], [18]. After 28 DAA, the grain starts to desiccate and gradually attains physiological maturity at about 42 DAA [14].

Microarray is a useful technique for accurate and high throughput gene expression analysis, though it is relatively less sensitive in detecting rare transcripts [19], [20]. Microarray technology has been extensively utilized to examine transcriptome related to grain development in wheat [14], [21], [22], [23], [24], [25], [26], [27]. We used Affymetrix GeneChip Wheat Genome Arrays to examine the transcriptome changes in developing grains of two genotypes, IITR26 and WL711, at 14 and 28 DAA. The study identifies several transcripts showing significant differential expression during grain filling, which could be candidate genes for mineral accumulation in grains at a higher level. The data set can be further examined for understanding the regulation of mineral accumulation and sequence polymorphism in the candidate genes.

## Materials and Methods

### Plant material

The two wheat genotypes, a wheat cultivar (*T. aestivum* Cv.WL711) and a landrace (*T. aestivum* L. IITR26), with contrasting levels of minerals in grain tissues [11], were grown in the experimental field of National Agri-Food Biotechnology Institute (NABI), SAS Nagar (Mohali) Punjab, India (310 m above sea level; Latitude 30° 47' North; Longitude 76° 41' East). The ears of each genotype were tagged at anthesis, in three replicates. The spikes were harvested at 14 and 28 DAA, frozen in liquid nitrogen immediately after harvesting and stored at −80°C for RNA extraction.

### Mineral concentration analysis

About 25 seeds of 14 DAA and 28 DAA were lyophilized (VirTis, sentry 2.0, USA) and crushed in a clean mortar - pestle. Approximately 100 mg powder was used for acid digestion in the Microwave Reaction System (Mars 6, CEM Corporation, USA). Mineral concentration was estimated in the digested samples using inductively coupled plasma mass spectrometry (ICP-MS; 7700×AgilentTechnologies, Santa Clara, CA), following standard protocol.

### RNA preparation

For RNA extraction, developing grains were extracted from the first and second floret of spikelet from the middle portion of the ear. Three grains were taken for RNA extraction in each sample. Total RNA was extracted using TRIzol (Invitrozen) and then purified with RNeasy Plant Mini Kit (Qiagen) following the manufacturer’s protocol. On-column DNase (Qiagen) digestion was performed as instructed in the manual. RNA integrity was determined using the RNA 6000 Nano assay bioanalyzer (Agilent).

### Microarray hybridization and data analysis

Double stranded cDNA synthesis, *in vitro* transcription to synthesize biotin labeled aRNA, purification and fragmentation of aRNA, and hybridization of arrays were performed according to Affymetrix technical manual. The Affymetrix GeneChip Wheat Genome Array contains 61,290 probe sets, representing about 25 K unigenes. The hybridized chips were washed, stained and scanned using the GeneChip scanner to generate the CEL files.

**Figure 1:**
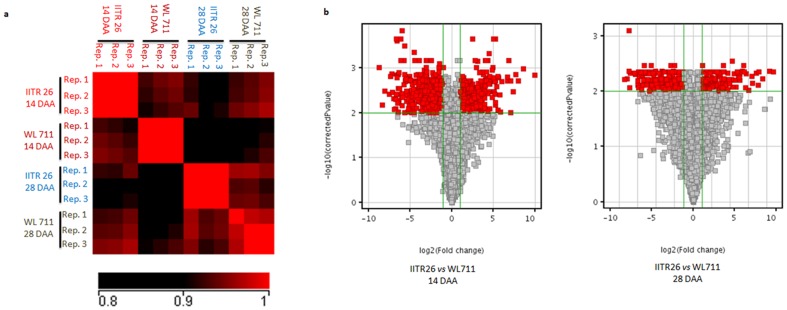
Differentially expressed transcripts at 14 and 28 DAA. (a) Correlation plot represents the pairwise correlation between biological replicates of the samples (b) Volcano plots represents the differentially expressed transcripts, satisfying the criteria of p≤0.01.

The CEL files were imported into GeneSpring GX v12 (Agilent Technologies). Signal intensities were recorded for all the 61,290 probe sets. The data has been deposited at NCBI (http://www.ncbi.nlm.nih.gov), with accession number GSE56032. The signal intensities were normalized by using Robust Multi-array Average (RMA) algorithm [28]. The Principal Component Analysis (PCA) in GeneSpring GX v12 established that the three biological replicates were located close to one another. The high correlation coefficient was observed among the three replicated samples, indicating less genetic background noise (Figure 1a). To correct the variability in the normalized expression values, the probe sets with coefficient of variation <50% were retained, and the rest were discarded. One-way analysis of variance (ANOVA, p≤0.01) was done with multiple testing correction algorithms using the Benjamini Hochberg approach with false discovery cut off, q ≤0.01. Volcano plot with *t-*test unpaired computation was performed (p≤0.01), and the transcripts showing statistically significant expression difference of two fold (*log2*) or above were selected for further analysis (Figure 1b). A *log2* ratio = 1 indicates a change in the expression of two fold.

### Functional annotation and gene ontology analysis

Functional annotation of the differentially expressed probe sets (with *log2* fold change of ≥2 at 14 and/or 28 DAA) was done by employing HarvEST Assembly: XW Affymetrix Wheat 1 version 1.59 (www.harvest.ucr.edu). Blast2GO annotation of the differentially expressed transcripts was performed for each probe set query sequence to enrich the annotation and to generate the combined graph of biological process. Hierarchical clustered heat map was produced for the differentially expressed transcripts, using MEV (version 4.6.2) [29]. Enrichment of Gene Ontology (GO) terms in significantly differentially expressed genes (≥2 *log2* fold change) was evaluated using AgriGO analysis tool (http://bioinfo.cau.edu.cn/agriGO) with Fisher tests and Bonferroni multiple testing correction (P<0.05) [30].

### In silico analysis of wheat transcripts

The differentially regulated transcripts were categorized by using MapMan software version 3.6.0RC1 (http://mapman.gabipd.org/web/guest/mapman). Spatial and temporal distribution pattern of the differential transcripts was examined at Genevestigator (https://www.genevestigator.com/gv/plant.jsp) using expression data of 1532 samples in *T. aestivum* microarray database of Affymetrix.

### Quantitative real time PCR

Total RNA was extracted from the developing grains (14 DAA) of the two genotypes (IITR26 and WL711), in three biological replicates. First strand cDNA was synthesized by using SuperScript III First Strand Synthesis Kit (Life Technologies). Primers were designed using Primer Express software. Quantitative real time PCR was performed in three biological replicates using SYBR Green (Qiagen, USA) fluorescence dye and analyzed by 7500 Fast Real-Time PCR System (Applied Biosystems). The qRT-PCR was performed using gene specific primers (Table S1 in File S1), and analyzed as described previously [31]. The wheat 18S gene was used as the internal control to normalize the expression data.

## Results and Discussion

### Mineral concentration

**Figure 2:**
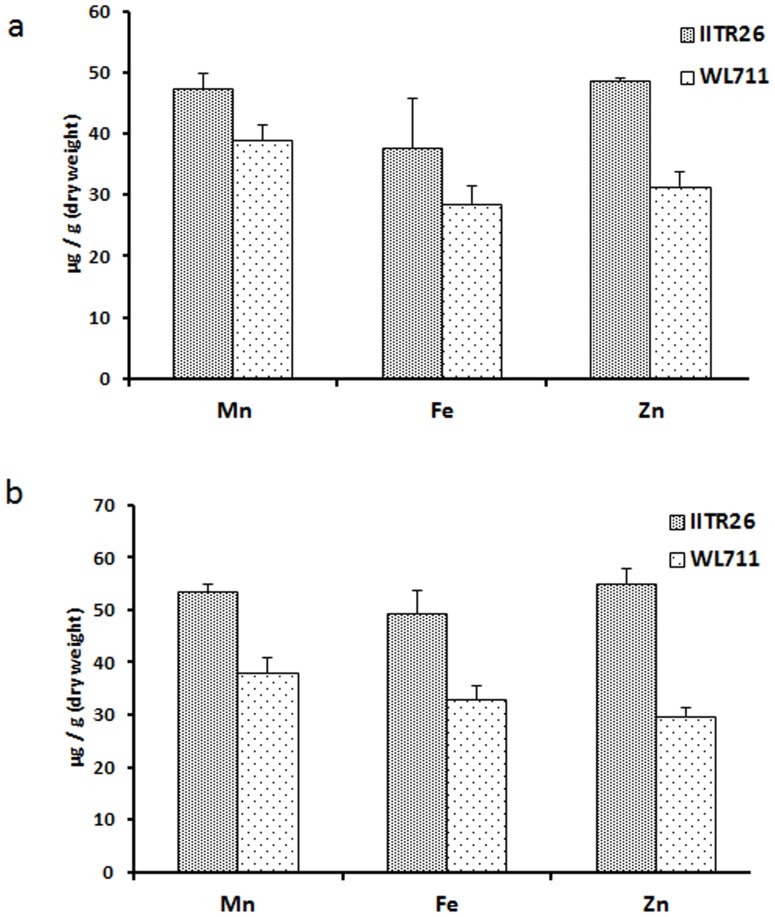
Concentration of micronutrients (Fe, Zn and Mn) in developing grains of IITR26 and WL711 of (a) 14 DAA and (b) 28 DAA.

The two genotypes, IITR26 and WL711, were analyzed for difference in mineral concentration in developing grains. The concentration of nutritionally important micronutrients, such as iron, zinc and manganese, was found significantly higher in the developing grains of IITR26, as compared to WL711 (Figure 2). This is in agreement with our previous reports which show a comparatively higher level of accumulation of minerals in the grain tissues of IITR26 [10], [11].

### Comparative transcriptional profiling and data analysis

**Figure 3:**
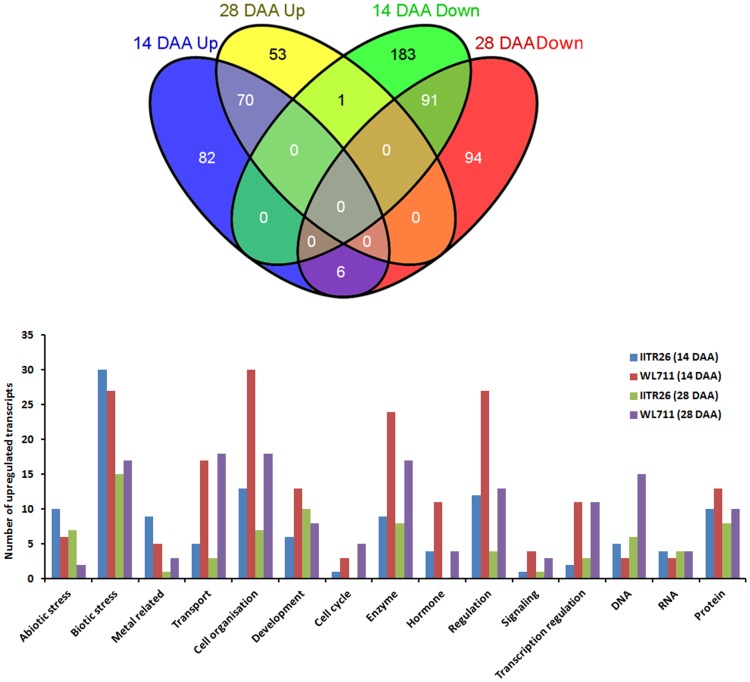
Differentially expressed transcripts with ≥2 *log2* fold change expression difference at p≤0.01, between IITR26 *vs.* WL711. (a) Venn diagram shows the total number of the differentially expressed transcripts and overlap at 14 and 28 DAA (b) Differentially regulated transcripts in biological and functional MapMan BINs.

**Figure 4:**
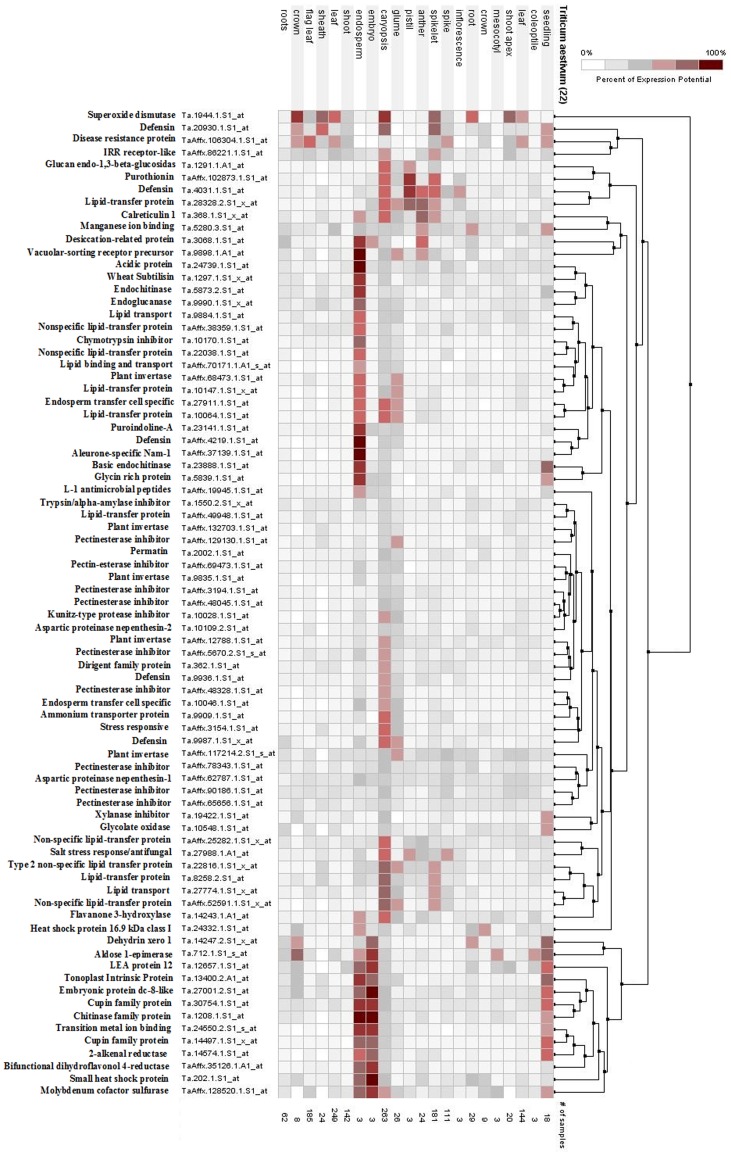
A heat-map of 170 transcripts listed in tables 3 and 4. The heat map shows the expression potential of the probe sets in 22 anatomical plant parts (Seedling, coleoptiles, leaf, shoot apex, mesocotyl, crown, root, inflorescence, spike, spikelet, anther, pistil, glume, caryopsis, embryo, endosperm, shoot, leaf, sheath, flag leaf, crown, roots), based on the previously reported 1532 experiments listed in Genevestigator (https://www.genevestigator.com). In the heat map, ‘Crown’ and ‘Root’ labeled at 6^th^ and 7^th^ column, respectively, represent the organs of seedlings.

Transcriptome analysis of developing grain was compared between two wheat genotypes (IITR26 and WL711). A total of 433 and 315 probe sets (with *log2* fold change of ≥2 at p≤0.01) were identified as differentially expressed between IITR26 *vs* WL711 on 14 and 28 DAA, respectively (Figure 3a). Putative gene function was assigned to each probe set (Table S2 in File S1) using public databases as mentioned in materials and methods. A total of 149 differentially regulated probe sets could not be assigned to any function, out of them 56 were common between 14 and 28 DAA. Among these, the GO enrichment patterns show disproportionately represented transcripts of genes involved in the biological process of response to stress, polysaccharide metabolism, multi-organism processes, and the biological function of nutrient reservoir and hydrolase activities in IITR26 developing grains (Table 1). In the developing grains of WL711, the GO biological processes- lipid transport, DNA replication, DNA metabolic process, starch metabolic process, and the functions- lipid binding, enzyme regulation, hydrolase and peptidase inhibitor activities were over-represented (Table 2). The functionally annotated transcripts were categorized into 15 bins by using MapMan software (Figure 3b) to understand global changes in developing grains of the two contrasting genotypes. Hierarchically clustered heat maps were produced for the 580 differentially expressed transcripts (Figure S1 in File S1). Meta-analysis indicated spatial and temporal distribution of the differentially regulated probe sets, assigned to putative locations of 22 anatomical plant parts based on their expression potential in the previously reported 1532 *T. aestivum* microarray (Affymetrix) experiments listed in Genevestigator (https://www.genevestigator.com). Several transcripts were identified showing predominant expression in grain tissues, as compared to other plant organs (Figures 4, S2 in File S1).

**Table 1:** Enrichment of GO terms in 282 genes up-regulated (≥2 *log2* fold) in developing grains of IITR26 during 14 and/or 28 DAA.

GO term	Ontology	Description	Contingency	P-value
GO:0044036	P	Cell wall macromolecule metabolic process	5, 85, 110, 34562	1.30E-05
GO:0051704	P	Multi-organism process	5, 366, 110, 34281	0.008
GO:0006950	P	Response to stress	12, 1726, 103, 34647	0.013
GO:0005976	P	Polysaccharide metabolic process	5, 455, 110, 34192	0.019
GO:0045735	F	Nutrient reservoir activity	5, 202, 110, 34445	0.00064
GO:0016798	F	Hydrolase activity, acting on glycosyl bonds	7, 430, 108, 34217	0.00065
GO:0004553	F	Hydrolase activity, hydrolyzing O-glycosyl compounds	5, 386, 110, 34261	0.0098

Key: P, biological process; F, molecular function.

Contingency denotes the number of genes in input list from the GO term, the number of genes on microarray from the GO term, the number of genes from input list not from the GO term, and number of genes on microarray not from the GO term.

**Table 2:** Enrichment of GO terms in 466 genes up-regulated (≥2 *log2* fold) in developing grains of WL711 during 14 and/or 28 DAA.

GO term	Ontology	Description	Contingency	P-value
GO:0006869	P	Lipid transport	5, 113, 210, 34534	0.00085
GO:0006260	P	DNA replication	6, 220, 209, 34427	0.0029
GO:0006259	P	DNA metabolic process	10, 625, 205, 34022	0.0064
GO:0009791	P	Post-embryonic development	6, 259, 209, 34388	0.0062
GO:0006073	P	Glucan/oligosaccharide/starch metabolic process	7, 352, 208, 34295	0.0072
GO:0030234	F	Enzyme regulator activity	8, 270, 207, 34377	0.00035
GO:0004553	F	Hydrolase activity, hydrolyzing O-glycosyl compounds	9, 386, 206, 34261	0.00083
GO:0004866	F	Endo-peptidase inhibitor activity	5, 121, 210, 34526	0.0011
GO:0008289	F	Lipid binding	6, 278, 209, 34369	0.0086

Contingency and key as described in Table 1.

### Candidate genes involved in the transport and accumulation of minerals and other solutes

Transcripts related to metal homeostasis showed differential expression levels in the developing grains of the two genotypes, IITR26 and WL711 (Table 3). Metallothionein (MT) was up-regulated in IITR26 developing grains as compared to WL711. MT is a cystein rich protein having the capacity to bind with metals through its thiol group. It plays an essential role in metal ion homeostasis, transport and storage in the cell [32], [33], [34]. In the seed, its abundance has been reported in aleurone and embryo [35], whereas endosperm shows comparatively less expression. This coincides with aleurone and embryo being hot spots for mineral localization [10]. Elevated transcription of Manganese ion and Transition metal ion binding proteins was observed in IITR26, mainly during 14 DAA (Table 3). Transition metal binding protein, an orthologue of Farnesylated protein 3, is involved in executing metal ion homeostasis and, biotic and abiotic stress tolerance [34]. Its expression has been recorded mostly in seed tissues (Figure 4).

**Table 3:** Differentially expressed transcripts involved in the transport and accumulation of minerals and other solute.

Probesets	Putative gene function	*Log2* Fold change (IITR26 *vs* WL711)		Corrected p-value
		14 DAA	28 DAA	
Ta.5280.3.S1_at	Manganese ion binding	2.01	0.14	1.65E-04
Ta.24550.2.S1_s_at	Transition metal ion binding	2.18	−0.52	2.20E-05
Ta.28347.1.S1_s_at	Metallothionein	3.56	2.39	1.86E-05
Ta.12657.1.S1_at	LEA protein 12	3.96	1.10	1.33E-05
Ta.6822.1.S1_at	Cinnamoyl-CoA reductase	2.54	0.60	4.83E-06
TaAffx.35126.1.A1_at	Bifunctional dihydroflavonol 4-reductase flavanone 4-reductase	3.71	0.73	1.30E-05
Ta.8881.1.S1_at	Dihydroflavonol-4-reductase	2.12	−0.29	1.57E-04
Ta.14243.1.A1_at	Flavanone 3-hydroxylase	2.35	−2.20	2.11E-04
TaAffx.37139.1.S1_at	Aleurone-specific Nam-1	2.00	0.15	1.10E-04
Ta.10548.1.S1_at	Glycolate oxidase	−3.13	−3.16	7.35E-05
Ta.368.1.S1_x_at	Calreticulin 1	−2.08	−0.99	6.33E-05
Ta.28543.1.A1_at	Protein phosphatase type 2c	−2.13	−1.92	4.75E-05
Ta.3862.1.S1_at	Probable protein phosphatase	−5.25	−1.13	6.69E-06
Ta.771.1.S1_at	Nicotianamine sythase 3	−0.19	−2.49	3.94E-05
Ta.556.2.A1_at	GDSL-like lipase/acylhydrolase	−7.16	−2.09	6.46E-06
Ta.8258.2.S1_at	Lipid-transfer protein (LTP) precursor	−5.09	−0.98	6.46E-06
Ta.28328.2.S1_x_at	Lipid-transfer protein (LTP) precursor	−4.89	−0.58	2.84E-05
Ta.27774.1.S1_x_at	Lipid transport	−3.71	−0.19	1.33E-05
Ta.9603.1.S1_s_at	Aquaporin NIP1	−3.37	−1.82	4.53E-05
Ta.1345.3.S1_x_at	Lipid-transfer protein (LTP) precursor	−3.21	−0.02	3.17E-05
Ta.9909.1.S1_at	Ammonium transporter protein	−2.05	−3.44	1.97E-04
Ta.11519.1.A1_at	Lipid transport	−3.21	−0.98	0.00128
Ta.22816.1.S1_x_at	Type 2 non-specific lipid transfer protein	−3.13	−2.07	4.83E-06
TaAffx.25282.1.S1_x_at	Non-specific lipid-transfer protein	−2.91	0.11	2.49E-05
Ta.7436.2.S1_at	Lipid-transfer protein (LTP) precursor	−2.86	−1.12	1.08E-04
Ta.10201.1.S1_x_at	Lipid-transfer protein (LTP) precursor	−2.84	−0.58	2.13E-05
TaAffx.70171.1.A1_s_at	Lipid binding and transport	−1.15	−6.94	3.11E-05
Ta.9884.1.S1_at	Lipid transport	−1.71	−6.81	6.77E-06
Ta.10147.1.S1_x_at	Lipid-transfer protein LTPL31	0.23	−6.00	1.93E-05
TaAffx.38359.1.S1_at	Nonspecific lipid-transfer protein	0.65	−5.64	6.01E-06
Ta.3605.2.S1_a_at	Lipid-transfer protein LTPL39	1.28	−5.43	1.05E-05
Ta.22038.1.S1_at	Nonspecific lipid-transfer protein	1.35	−5.28	1.33E-05
Ta.8141.3.S1_a_at	Lipid-transfer protein LTPL42	−0.41	−4.09	1.06E-04
Ta.14034.1.A1_at	Lipid-transfer protein LTPL128	1.06	−3.38	7.95E-05
Ta.23917.3.S1_x_at	Non-specific lipid-transfer protein	−2.95	−3.30	7.48E-05
Ta.13168.1.S1_a_at	Lipid-transfer protein LTPL38	−0.81	−3.27	1.87E-04
Ta.30659.1.S1_at	Aluminum-activated malate transporter	−1.52	−2.90	3.00E-04
Ta.25314.1.A1_s_at	Phosphoenolpyruvate phosphate translocator	−1.74	−2.73	3.89E-04
Ta.28055.2.S1_at	Lipid transport, lipid binding	−0.56	−2.47	4.83E-06
Ta.25583.1.S1_at	Lipid-transfer protein precursor	−1.07	−2.39	9.06E-06
Ta.10064.1.S1_at	Lipid-transfer protein precursor	−0.04	−2.13	4.88E-05
TaAffx.49948.1.S1_at	Lipid-transfer protein precursor	−2.44	−1.19	3.12E-05
Ta.28067.1.S1_x_at	Nonspecific lipid-transfer protein	−2.35	−3.10	4.64E-04
TaAffx.52591.1.S1_x_at	Non-specific lipid-transfer protein	−2.23	−0.24	2.13E-04
TaAffx.97181.1.S1_s_at	Lipid-transfer protein precursor	−2.06	−0.01	6.75E-04
Ta.13400.2.A1_at	Tonoplast Intrinsic Protein (TIP3)	2.98	0.30	1.08E-04
Ta.2895.1.S1_x_at	Plasma membrane intrinsic protein (PIP1)	3.41	1.97	0.01438
Ta.9898.1.A1_at	Vacuolar-sorting receptor precursor	5.21	3.20	1.20E-05
Ta.27916.1.A1_x_at	Transmembrane amino acid transporter	−3.41	0.34	1.23E-04
TaAffx.128497.1.S1_at	Amino acid transporter	0.56	2.28	4.31E-05
TaAffx.22704.1.S1_at	Amino acid transporter	2.68	0.39	0.0105
Ta.29726.2.A1_at	Protein transport protein (SecE/Sec61)	5.51	5.04	5.04E-06

A transcript of the NAM-1 gene was found up-regulated in developing grains of IITR26 during grain filling. RNAi mediated suppression of NAM in wheat resulted in lower grain mineral and protein concentrations [36], [37]. Further, expression of the probe set was found limited to endosperm in meta-analysis (Figure 4). Seed specific NAM-1 could be one of the candidate genes which play role in mineral accumulation in wheat, during grain filling.

Late embryogenesis abundant (LEA) proteins play crucial role in conferring tolerance to metal stress [38], [39]. LEA proteins are suggested to be involved also in sequestering divalent metals, and micronutrient trafficking in phloem tissues [40], [41]. Enhanced expression of LEA-12 could facilitate higher level of mineral accumulation in the grains of IITR26.

Elevated expression of lignin biosynthesis related genes have been observed, mainly during metal stress, in metal hyper-accumulator plants [42]. The up-regulated Cinnamoyl CoA reductase gene, an enzyme of the lignin branch biosynthetic pathway, presumably provides metal tolerance in developing grains of IITR26. Up-regulation of Flavonoid biosynthetic pathways genes has been notified under metal and biotic stresses [43]. Flavonoids, such as quercetin, have capacity of chelation of transitional metals [44]. Flavanone 3-hydroxylase has sites for putative iron binding [45]. Genevestigator analysis revealed the expression of Flavanone 3-hydroxylase and Dihydroflavonol-4-reductase genes largely in seed tissue (Figure 4). Their up-regulation, during early grain filling stage in IITR26, could be helpful in favorably accumulating more transition metals [46].

In WL711 developing grains, transcripts for Protein phosphatase 2c-PP2c were significantly up-regulated (Table 3). PP2c is involved in the regulation of cation transporters and membrane polarization, influencing ion homeostasis in plant cells during stress and during phloem transport [47], [48]. The Calreticulin-1 gene was up-regulated in WL711 developing grains, and its presence has been found mostly in seed tissues (Figure 4). It codes for multifunctional protein that binds Ca ions (as a second messenger) with low affinity, but high capacity, and its role has been demonstrated in drought and metal stress tolerance [49]. A probe set representing Glycolate oxidase gene, an important player in oxalate biosynthesis, was significantly up-regulated in the developing seeds of WL711 (Table 3). Oxylate has been notified to play important role in calcium regulation and metal detoxification, and its access reduces calcium bioavailability [50]. Nicotianamine (NA) is a metal chelator. NA is synthesized by trimerization of three molecules of S-adenosyl-Lmethionine by nicotianamine synthase (NAS). The role of NAS-3 has been reported in mineral homeostasis in developing organs in maize [51]. Induced expression of NAS3 has been recorded during excessive mineral, while expression is down regulated under mineral deficient condition [52]. The up-regulation of NAS-3, at later grain filling stage (Table 3), could be indicative of mineral sufficiency within the grain of WL711, as compared to IITR26.

In wheat, aleurone is a single cell layer storage tissue which accumulates minerals, phytic acid and proteins in globoids or protein storage vacuoles (PSV), within the cell [53]. High resolution X-ray micrograph of aleurone cell revealed the well defined edges of globoids, surrounded by a membrane, tonoplast [54]. Aquaporins, localized in the tonoplast of PSVs [55], [56], facilitate transmembrane transport of water and solutes, and provide cellular adaptation to metal stress by water homeostasis [57], [58]. About three fold increased expression of aquaporin- Tonoplast intrinsic protein (TIP-3) was recorded in IITR26 developing grains (Table 3). Meta-analysis showed its abundance in seed tissues as compared to other organs (Figure 4). Some other aquaporins, Plasma membrane intrinsic protein (PIP-1) and Nodulin 26-like intrinsic protein (NIP-1), play an important role in osmo-adaptation and homeostosis of cells to facilitate transport of water and phloem derived nutrients to grain tissues [59], [60]. However, NIP-1 has been notified as less efficient in transporting solute and ions [61]. Enhanced expression of TIP-3 and PIP-1 could promote higher level of mineral sequestration in IITR26 grains (Table 3). The aleurone globoids or PSVs store large number of organelle-specific proteins, synthesized by endosplasmic reticulum associated ribosomes and transported through vacuolar sorting [62]. Vacuolar sorting receptor-1 (VSR-1) is involved in sorting of proteins in PSV [63]. In meta-analysis, the predominance of VSR-1 was observed in endosperm (Figure 4). The enhanced expression of VSR-1 in IITR26 (Table 3) could be suggestive of higher storage activity [64] in IITR26 grains.

Several probe sets related to lipid transport and binding (LTP family protein) were significantly up-regulated in the developing grains of WL711 (Table 3). The expression of many of them has been recorded predominant in grain, as compared to other anatomical part (Figure 4). The enhanced expression of GDSL esterases/lipases, that catalyze hydrolysis of ester bonds- primarily in triglycerides, indicates relatively more active lipid metabolism [65] in WL711 developing grains. LTPs regulate lipid transport, homeostasis and lipid-mediated cellular processes [66]. Within grain, several LTPs have been observed up-regulated in aleurone cells around the embryo, scutellum and vascular bundle of the embryo, resulting maximum accumulation of lipids in embryo in grains [67], [68]. Their role in providing protection to embryo from pathogen attack has been speculated [65], [67], [68]. Unlike WL711, transporters for amino acid and protein (SecE) [69] were expressed at higher level in IITR26 during grain filling (Table 3). This is in agreement with previous reports showing significant correlation between grain protein and micronutrient content [15], [70], [71]. The results also coincide with our observation of proportional association between elemental images of sulphur (possibly sulphur-rich peptides) and iron in cross-sections of the two contrasting genotypes [10].

### Candidate genes representing stress responses and metal tolerance

**Figure 5:**
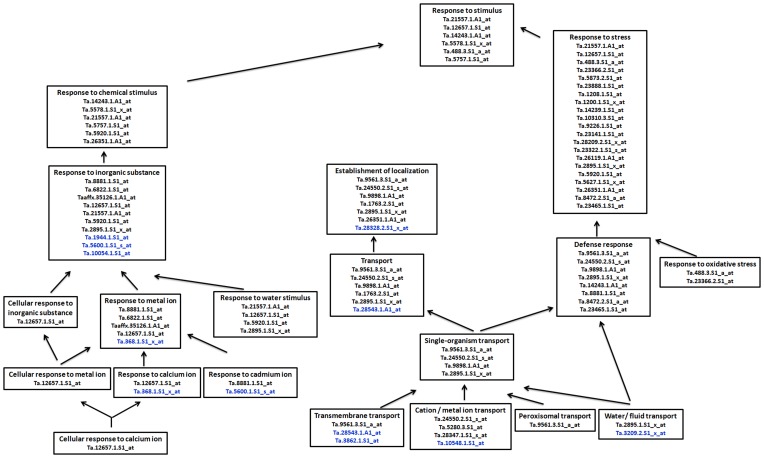
Distribution of differentially up-regulated (IITR26 *vs.* WL711; ≥2 *log2* fold change; p≤0.01) transcripts related to metal accumulation and stress response in IITR26 (probe sets in black) and WL711 (probe sets in blue) at 14 DAA. Data is extracted from Blast2GO results of combined graphs (Biological Processes). The annotation of the probe sets is given in tables S3.

Blast2GO results of combined graphs distributed the differentially expressed transcripts in different levels of biological processes, mapping the transcripts related with metal/cation transport, localization and responses etc. The results revealed transcripts representing stress response to metal ion activity in developing grains of IITR26, such as peroxins (Ta.9561.3.S1_a_at), VSR-1 (Ta.9898.1.A1_at), PIP-1 (Ta.2895.1.S1_x_at), Ankyrin repeat protein (Ta.23465.1.S1_at) and Flavonoid (Ta.8881.1.S1_at) (Figure 5). Peroxisome biogenesis proteins (peroxins) are involved in docking cargo-receptor complexes at the peroxisomal membrane [72]. Several studies revealed essential role of peroxins in defense mechanisms conferring resistance against pathogen attack [73], [74]. VSR-1 is a candidate gene controlling the defense network, and its up-regulation has been reported in metal tolerant and disease resistant plants [75], [76]. Involvement of plasma membrane aquaporins (PIP-1) has been discussed in metal homeostasis and defense response in plants [77], [78], [79]. Flavonoids enhance protective abilities of the plant against biotic stresses [80], [81]. Ankyrin repeat proteins have been reported to mediate metal tolerance and enhanced disease resistance in plants [82], [83].

**Figure 6:**
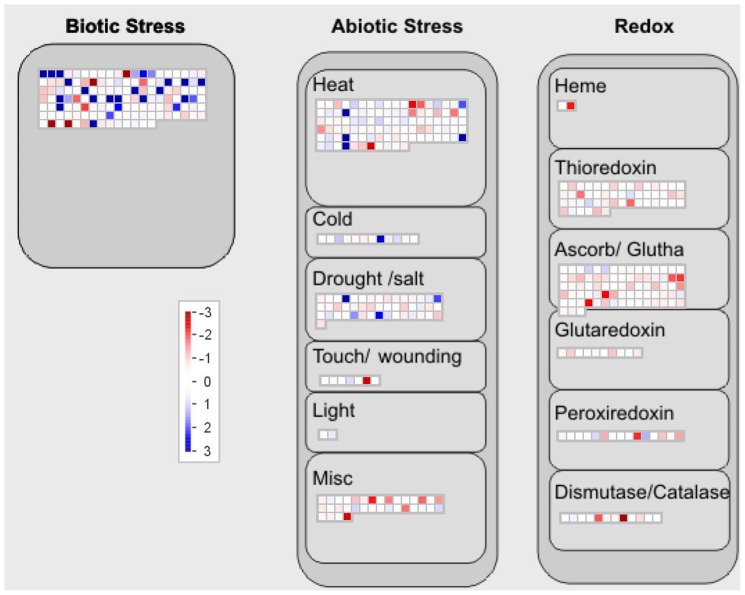
Differentially expressed transcripts, satisfying the criteria of p≤0.01 (IITR26 *vs.* WL711) at 14 DAA, identified in ‘cellular response overview’ using MapMan software version 3.6.0RC1. The *log2* fold change in the transcript levels were used for the analysis.

Several genes associated with abiotic and biotic stress were found differentially regulated in developing grains of both the genotypes (Table 4), presumably having coordination among the growth, maintenance and stress during seed development [84]. For example, the transcripts for β-purothionin, cysteine-rich proteins, and Defensin, up-regulated in WL711, are known to play significant role in protecting wheat grains from microbial pathogens [68], [85]. In IITR26 developing grains, transcripts of Puroindolin-A (PINa), a cystine- and tryptophan-rich protein, was significantly predominant. However, the probe set for PINb did not pass the quality criteria. A relationship has been incurred between grain hardness and puroindoline content [86]. However, both the genotypes, IITR26 and WL711, exhibit similar grain hardness indices (∼90) [12], [87], [88]. This indicates contribution of PINa in grain related traits, other than hardness. Role of PINa protein has been established in plant defense as antimicrobial molecule [85]. Transcripts of many other antimicrobial molecules, Chitinase, Glycin rich proteins, Wheatwin, VAMP, Bowman-Birk and Kunitz-type protease inhibitor, were significantly up-regulated in the developing grains of IITR26 (Table 4). A positive correlation has been inferred between degree of biotic stress resistance and glucan synthase activity, on the contrary glucanase activity is negatively correlated [89]. The enhanced glucan synthase activity and reduced glucanase activity in IITR26 developing grains further corroborates enhanced resistance to pathogen attack in IITR26 (Table 4). Several fold reduction in superoxide dismutase activity in the developing grain of IITR26 (Table 4), could be indicative of less reactive oxygen species (ROS) formation [90]. Mampman analysis of cellular response overview reflected up-regulation of redox related transcripts in WL711 during grain filling (Figure 6). This is in agreement with the previous report of induced redox state in susceptible wheat as compared to the biotic stress tolerant line [91]. Meta-analysis determined dominant expression potential of several stress related transcripts in grain tissues (Figure 4), as compared to other plant organs, suggesting their role in seed development. The similarity search in Genevestigator, using the differentially expressed transcripts, revealed perturbations in which spikelet samples of wheat genotypes, with stress resistant (CS-7EL) and susceptible (CS) backgrounds (GEO accession GSE21386), have been compared (Figure S3 in File S1). The results coincide with the previous reports examining interactions between metal ion excess and biotic stress factors [92], [93], and the responses to excess metal ions resemble to that of biotic and abiotic stresses [94], [95]. Our results support the elemental defense hypothesis which suggests a crosstalk between metal accumulation and biotic and abiotic stress responses [96], [97], [98], [99]. However, it needs to be investigated and validated in other wheat genotypes, with contrasting levels of minerals in grains, if the crosstalk substantiates the enhanced level of mineral sequestration in grains.

**Table 4:** Differentially expressed transcripts representing metal tolerance and stress responses.

Probesets	Putative gene function	*Log2* Fold change (IITR26 *vs* WL711)		Corrected P-values
		14 DAA	28 DAA	
TaAffx.102873.1.S1_at	Purothionin	−7.33	−4.84	3.17E-05
Ta.20930.1.S1_at	Defensin	−7.22	−1.22	9.62E-05
Ta.9987.1.S1_x_at	Defensin Tk-AMP-D4	−4.03	−2.20	3.83E-04
Ta.9936.1.S1_at	Defensin Tk-AMP-D5	−3.33	−0.57	1.09E-05
Ta.4031.1.S1_at	Defensin	−2.69	−0.06	1.06E-04
TaAffx.4219.1.S1_at	Defensin	2.14	−0.53	0.001154
Ta.362.1.S1_at	Dirigent family protein	−6.75	−0.15	4.58E-05
Ta.7883.1.S1_x_at	Dirigent like protein	−3.76	0.00	5.30E-06
Ta.1944.1.S1_at	Superoxide dismutase	−6.61	−5.97	3.94E-06
Ta.5600.1.S1_s_at	Salt tolerance protein	−5.87	−0.31	7.86E-05
Ta.27988.1.A1_at	Salt stress response/antifungal	−2.41	−0.19	4.05E-04
Ta.27725.1.S1_at	Salt stress-induced hydrophobic peptide ESI3	2.77	0.30	7.10E-05
Ta.2002.1.S1_at	Permatin, thaumatin, osmotin 34	−5.64	−0.76	1.33E-05
Ta.5878.1.S1_at	Aldo/keto reductase family protein	−4.45	−5.09	1.22E-05
TaAffx.62787.1.S1_at	Aspartic proteinase nepenthesin-1	−3.52	−0.16	8.64E-05
Ta.10109.2.S1_at	Aspartic proteinase nepenthesin-2	−3.26	−2.00	3.25E-04
Ta.5072.1.S1_at	Wound induced protein	−3.26	−4.48	7.06E-06
Ta.19041.1.S1_at	Wound-responsive family protein	−2.74	−1.69	0.00163
TaAffx.3154.1.S1_at	Stress responsive	−3.25	−0.56	3.94E-06
Ta.9967.1.S1_at	Cytochrome P450	−2.88	−1.16	5.04E-06
Ta.3382.1.S1_at	Cytochrome P450	0.29	−2.42	1.79E-05
Ta.21115.3.A1_s_at	Cytochrome p450 like	1.05	−2.89	0.005749
Ta.3361.3.S1_a_at	Pathogenesis-related Bet v I family protein	−2.64	−3.78	9.06E-06
Ta.3094.2.S1_at	GDP-mannose 3,5-epimerase 1	−2.51	−1.75	2.35E-04
Ta.28866.1.S1_at	Patatin-like protein	−2.49	0.21	2.36E-05
Ta.21001.1.S1_at	Gamma-glutamyltranspeptidase 1 precursor	−2.49	−0.63	5.47E-05
TaAffx.56549.1.S1_at	Beta-glucosidase	−2.40	−1.91	9.84E-04
Ta.18082.1.S1_at	Beta-glucosidase	−2.13	0.04	4.09E-04
Ta.4683.1.S1_at	Beta-glucosidase, exo-beta-glucanse	0.17	−2.27	3.92E-05
Ta.2834.1.S1_at	Glucan endo-1,3-beta-glucosidase	−2.39	0.30	4.01E-04
TaAffx.73807.1.A1_at	Glucan endo-1,3-beta-glucosidase	−0.21	−3.08	3.82E-05
Ta.9958.1.S1_at	Glucan 1,3-beta-glucosidase	−4.69	−0.04	2.06E-05
Ta.1291.1.A1_at	Glucan endo-1,3-beta-glucosidase GIV	−3.86	−0.41	4.83E-06
Ta.9990.1.S1_x_at	Endoglucanase	−0.07	−4.17	1.65E-05
TaAffx.117214.2.S1_s_at	Plant invertase/pectin methylesterase inhibitor	−6.70	−0.06	2.12E-06
TaAffx.68473.1.S1_at	Plant invertase/pectin methylesterase inhibitor	−6.64	−2.54	2.22E-05
TaAffx.12788.1.S1_at	Plant invertase/pectin methylesterase inhibitor	−5.63	−0.13	5.09E-06
Ta.27567.1.S1_at	Plant invertase/pectin methylesterase inhibitor	−5.48	−0.24	1.30E-05
TaAffx.132703.1.S1_at	Plant invertase/pectin methylesterase inhibitor	−4.91	−0.06	3.06E-05
TaAffx.65656.1.S1_at	Pectinesterase inhibitor domain containing protein	−4.57	0.15	4.83E-06
TaAffx.78343.1.S1_at	Pectinesterase inhibitor domain	−3.97	0.05	1.54E-05
TaAffx.3194.1.S1_at	Pectinesterase inhibitor domain containing protein	−3.06	−0.73	6.77E-06
TaAffx.48045.1.S1_at	Pectinesterase inhibitor domain containing protein	−3.02	−2.26	0.001093
TaAffx.69473.1.S1_at	Pectin-esterase inhibitor	−3.00	−2.45	2.36E-04
TaAffx.90186.1.S1_at	Pectinesterase inhibitor	−2.60	−0.44	1.13E-04
Ta.9835.1.S1_at	Plant invertase/pectin methylesterase inhibitor	−2.52	−3.62	1.47E-05
TaAffx.48328.1.S1_at	Pectinesterase inhibitor domain containing protein	−2.02	−1.35	1.15E-04
TaAffx.5670.2.S1_s_at	Pectinesterase inhibitor	−2.11	−2.61	3.79E-04
TaAffx.129130.1.S1_at	Pectinesterase inhibitor domain containing protein	−5.28	−4.77	1.05E-05
Ta.5969.3.S1_x_at	Heme-binding protein	−2.27	0.40	0.00568
TaAffx.86221.1.S1_at	IRR receptor-like serine threonine-protein kinase	−2.24	−0.31	0.00597
Ta.13256.2.S1_at	Thaumatin family domain containing protein	−2.12	−1.30	5.63E-05
Ta.10046.1.S1_at	Endosperm transfer cell specific PR9	−1.25	−7.16	5.09E-06
Ta.27911.1.S1_at	Endosperm transfer cell specific PR60	−0.17	−4.06	5.32E-05
Ta.1762.1.A1_at	Jasmonate-induced protein	−1.15	−2.75	4.99E-06
Ta.24739.1.S1_at	Acidic protein	−0.65	−4.21	7.63E-05
Ta.28539.1.A1_x_at	NAC domain-containing protein	−0.18	3.43	1.16E-05
Ta.5367.1.S1_s_at	NAC domain-containing protein 67	−0.66	3.28	1.19E-04
Ta.16815.1.S1_s_at	Tetratricopeptide repeat domain	0.01	2.01	2.20E-05
Ta.10170.1.S1_at	Chymotrypsin inhibitor WCI	0.06	−2.26	3.00E-05
Ta.14247.2.S1_x_at	Dehydrin xero 1	0.07	3.09	7.06E-06
Ta.8834.1.S1_x_at	Cysteine-rich repeat secretory protein 55 precursor	0.23	−2.63	3.34E-05
Ta.202.1.S1_at	Small heat shock protein	0.31	2.63	2.51E-04
Ta.712.1.S1_s_at	Aldose 1-epimerase	0.42	3.17	1.93E-05
TaAffx.10772.1.A1_at	Serine rich protein	0.46	2.79	5.80E-05
Ta.21508.1.A1_a_at	Oxidative Stress (OXS3)	0.61	2.02	6.07E-06
TaAffx.12591.1.S1_at	Disease resistance gene RAR1	2.05	1.49	1.94E-04
Ta.22179.1.A1_at	Disease resistance protein rga4	2.45	−0.44	0.007391
TaAffx.104814.1.S1_at	Disease resistance protein rga1-like	−2.62	−2.02	3.73E-05
TaAffx.106304.1.S1_at	Disease resistance protein NBS-LRR	0.14	2.05	1.78E-05
Ta.30674.1.S1_at	Peroxidase precursor	−2.38	0.88	1.36E-04
Ta.7262.1.A1_x_at	Peroxidase precursor	0.73	2.14	1.31E-04
Ta.9334.1.S1_s_at	Peroxidase precursor	1.32	−3.29	2.15E-04
Ta.488.3.S1_a_at	Peroxidase precursor	2.17	0.02	2.58E-04
Ta.23366.2.S1_at	Peroxidase precursor	2.98	7.14	6.85E-06
Ta.6572.2.S1_at	Peroxiredoxin Q	−2.22	−0.99	3.20E-05
Ta.9561.3.S1_a_at	Peroxisomes biogenesis proteins Peroxin Pex14	4.05	3.24	3.94E-06
Ta.28209.2.S1_x_at	BURP domain-containing protein RD22	2.07	0.95	0.00253
Ta.21557.1.A1_at	Senescence/dehydration associated protein	2.26	3.30	1.30E-05
Ta.6399.1.A1_at	Protease do-like 14	2.30	1.66	2.46E-04
TaAffx.128.1.S1_at	Chaperone protein dnaj 6-like	2.37	1.58	0.016101
Ta.27001.2.S1_at	Embryonic protein dc-8-like	2.46	0.25	2.12E-05
Ta.23322.1.S1_s_at	Thaumatin-like protein	2.56	1.88	2.55E-05
Ta.23465.1.S1_at	Ankyrin repeat family protein	2.57	1.81	0.009715
Ta.12273.1.A1_at	Ankyrin repeats	4.34	4.61	2.00E-05
Ta.8472.1.S1_at	Ankyrin repeat protein	4.21	3.81	1.40E-05
Ta.14574.1.S1_at	2-alkenal reductase	2.69	3.74	6.85E-06
Ta.14497.1.S1_x_at	Cupin family protein	2.72	−0.15	4.59E-05
Ta.30754.1.S1_at	Cupin family protein	2.99	−0.31	2.45E-05
Ta.26119.1.A1_at	3-isopropylmalate dehydrogenase	2.94	2.34	0.00429
Ta.3268.1.A1_at	F-box domain	2.05	1.86	0.009745
TaAffx.92919.1.A1_at	F-Box and Domain of Unknown Function Containing Proteins	2.12	0.55	5.93E-05
Ta.7304.1.A1_at	F-box domain containing protein	4.17	2.13	0.014434
TaAffx.64850.1.A1_at	F-box and FBD domain containing protein	4.56	4.80	3.94E-06
TaAffx.120564.1.A1_at	FDB-associated f-box protein at1g66310-like isoform x1	4.86	4.90	4.95E-06
Ta.5627.1.S1_x_at	VAMP protein	3.19	0.83	7.01E-05
Ta.5920.1.S1_at	ABA related (HVA22)	3.38	1.01	1.40E-05
Ta.10310.3.S1_at	ABA related	5.77	0.90	4.94E-05
Ta.5873.2.S1_at	Endochitinase	3.32	0.10	3.89E-05
Ta.23888.1.S1_at	Basic endochitinase	5.24	−0.16	1.68E-05
Ta.6952.1.S1_a_at	Chitinase	5.36	3.28	0.014148
Ta.1208.1.S1_at	Chitinase family protein precursor	8.00	2.02	3.94E-06
TaAffx.128520.1.S1_at	Molybdenum cofactor sulfurase family protein	3.43	0.30	2.59E-05
Ta.5257.2.S1_s_at	Low temperature and salt responsive protein	3.50	1.34	0.013306
Ta.3068.1.S1_at	Desiccation-related protein PCC13-62 precursor	3.56	0.18	6.99E-05
Ta.1550.2.S1_x_at	Trypsin/alpha-amylase inhibitor CMX1/CMX3	3.73	0.18	0.001797
Ta.1297.1.S1_x_at	Wheat Subtilisin	3.93	1.48	1.16E-04
Ta.20434.1.S1_at	Xylanase inhibitor	1.84	2.84	6.02E-04
Ta.19422.1.S1_at	Xylanase inhibitor	4.30	0.95	3.61E-05
Ta.1200.1.S1_x_at	Xylanase inhibitor	5.87	0.00	7.72E-05
Ta.26351.1.A1_at	Glucan synthase-like	4.46	0.33	7.77E-06
Ta.24332.1.S1_at	Heat shock protein 16.9 kDa class I	4.92	8.27	2.29E-04
Ta.20928.1.S1_at	Bowman-Birk inhibitor (BBI) gene	5.16	5.31	9.43E-06
Ta.9226.1.S1_at	Wheatwin	5.21	1.24	1.09E-05
Ta.10028.1.S1_at	Kunitz-type protease inhibitor	5.60	−0.71	3.92E-05
Ta.5839.1.S1_at	Glycin rich protein	7.06	−0.01	4.36E-05
TaAffx.19945.1.S1_at	L-1, precursor of antimicrobial peptides	7.36	1.59	3.76E-04
Ta.23141.1.S1_at	Puroindoline-A	9.99	7.51	5.30E-06

Pectin methylesterase, which catalyses the de-esterification of pectin, is regulated by pectin methylesterase inhibitor. Its role has been discussed in wounding, osmotic stress, senescence and seed development [100]. Significant up-regulation of several pectin methylesterase inhibitor related probe sets has been observed in WL711 (Table 4). Down regulation of pectin methylesterase inhibitor activity might inhibit de-esterification of pectin, which could result into reduced metal tolerance in the tissue [101].

The developing grains of IITR26 exhibited up-regulation of transcripts for F-box and NAC genes (Table 4). F-box proteins, apart from its role in seed development [102], provide tolerance during metal stress in plant tissues [103]. NAC proteins play a key role during stress signaling pathways and provide tolerance against various abiotic and biotic stresses [104]. Furthermore, the abscisic acid (ABA)/stress-induced proteins [105], such as HVA22 were expressed at higher level in IITR26 developing grains (Table 4).

The results are suggestive of IITR26 being comparatively more tolerant to stress during grain filling, which eventually could be a positive factor for metal accumulation.

### Quantitative RT-PCR and similarity search meta-analysis

**Figure 7:**
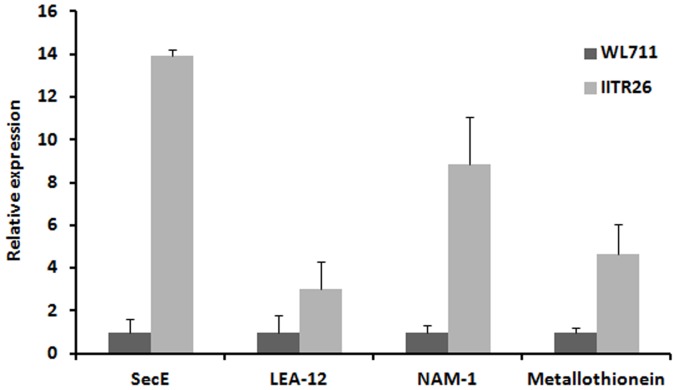
Quantitative RT-PCR analyses of a few candidate genes: Metallothionein, NAM-1, LEA-12, and Sec-E. Each bar indicates standard error in three biological replicates.

The relative fold change in expression of a few genes related to the transport and accumulation of metal was examined by real time PCR analysis (Figure 7). The expression of Metallothionein, NAM-1 and LEA-12, and protein, SecE, were found high in the developing grains of IITR26. The expression profile of these genes was in agreement with the microarray results. Hence, q-RT PCR results validated the microarray data, as reported in several published reports.

**Figure 8:**
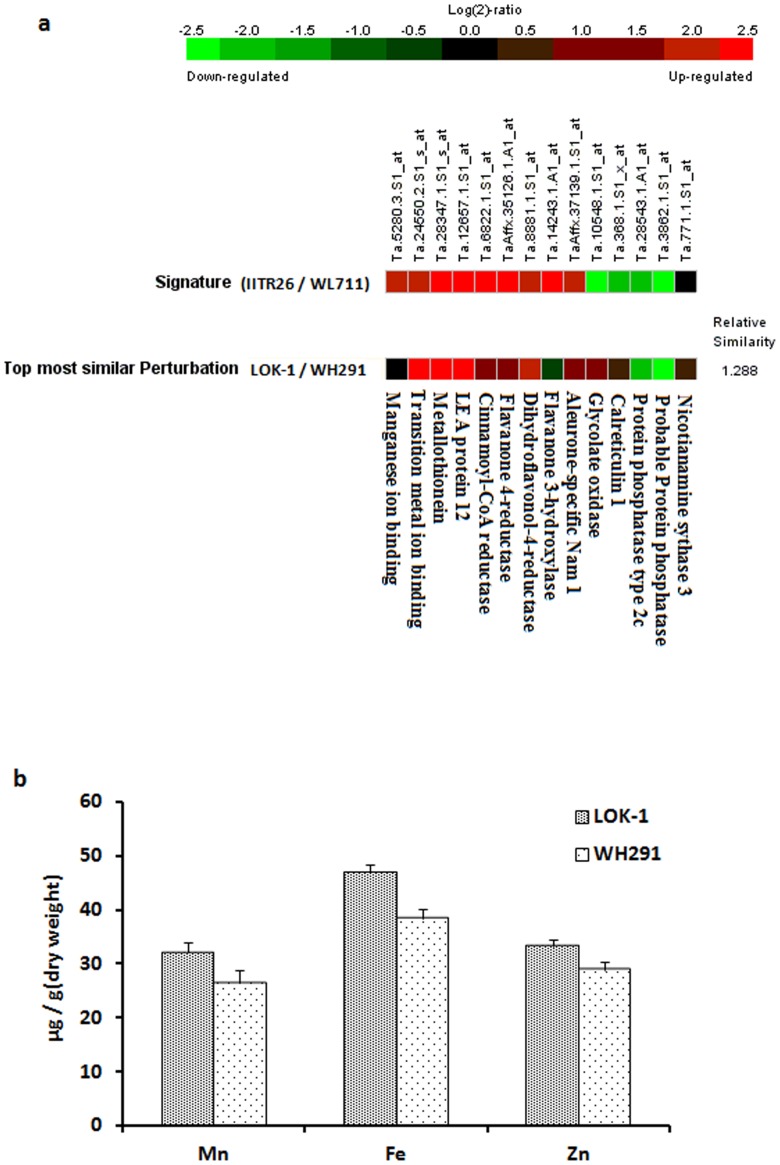
Similarity search meta-analysis and mineral concentration analysis. (a) The similarity search in Genevestigator, using the differentially expressed metal related transcripts in our data, revealed the top most perturbation comparing transcriptome between the developing grains of LOK-1 and WH291. (b) Concentration of micronutrients (Fe, Zn and Mn) in mature grains of LOK-1 and WH291.

Similarity search meta-analysis was performed against 1532 experiments listed in Genevestigator, by using the metal related transcripts differentially expressed between IITR26 and WL711 (Table 3). The perturbation showing maximum similarity with our data comprised the transcriptional comparison between the developing grains of two wheat genotypes: LOK-1 and WH291 (Figure 8a). The mineral concentration analysis revealed relatively higher concentrations of micronutrients in mature grains of LOK-1 than WH291 (Figure 8b), which coincides with up-regulation of the metal related transcripts in early-stage developing grains (Figure 8a), supporting our data.

## Conclusion

The microarray based comparative transcriptome profiling of developing grains of two genotypes contrasting for grain mineral concentration identified several differentially expressed genes. Prevalence of transcripts related to metal transport and accumulation, metal tolerance, vacuolar sorting receptor-1, aquaporins (TIP-3 and PIP-1), amino acid and protein transporters, lignin and flavonoid biosynthesis, and stress responses, was noticed in IITR26 during grain filling. For some of the differentially expressed probe sets, gene functions are yet to be determined (Table S2 in File S1). Thus, the differential expression analysis of microarray data revealed several candidate genes which may facilitate the elevated levels of minerals in the grains. However, the possibility of involvement of the differential transcripts in establishing other traits, for which the two genotypes differ, may not be denied. The differential expression data provided in this study would be useful for designing programs for functional genomics, exploration of sequence polymorphism in the candidate genes, and understanding other metabolic pathways common with mineral accumulation in wheat grains.

## Supporting Information

File S1 Contains the following files: **Figure S1.** Hierarchically clustered heat-map for 580 differentially expressed transcripts (IITR26 *vs.* WL711; ≥2 *log2* fold change; p≤0.01) at 14 and 28 DAA. **Figure S2.** A heat map of differentially expressed transcripts (IITR26 vs. WL711; ≥2 *log2* fold change; p≤0.01) at (a) 14 and (b) 28 DAA. The rest of the details are as given in figure 4. **Figure S3.** The similarity search in Genevestigator, using the differentially expressed transcripts (IITR26 vs. WL711; ≥2 *log2* fold change; p≤0.01) at 14 DAA revealed perturbations (top 5) in which spikelet samples of wheat genotypes, with stress resistant (CS-7EL) and susceptible (CS) backgrounds (GEO accession GSE21386), have been compared. **Table S1.** Gene-specific primers used for qRT-PCR. **Table S2.** Table S1 Differentially regulated probe sets with ≥2 *log2* fold change expression difference at p≤0.01, between IITR26 vs. WL711, and their putative gene function during 14 and 28 DAA. **Table S3.** Annotation of the probe sets mentioned in Figure 5.
(ZIP)
