# Supplementary material for: Comparative Transcriptional Profiling of Two Wheat Genotypes, with Contrasting Levels of Minerals in Grains, Shows Expression Differences during Grain Filling
Source: PLoS One. 2014 Nov 3;9(11):e111718. doi: 10.1371/journal.pone.0111718 (PMC4218811; doi:10.1371/journal.pone.0111718)
Supplement: File S1 — Contains the following files: Figure S1. Hierarchically clustered heat-map for 580 differentially expressed transcripts (IITR26 vs. WL711; ≥2 log2 fold change; p≤0.01) at 14 and 28 DAA. Figure S2. A heat map of differentially expressed transcripts (IITR26 vs. WL711; ≥2 log2 fold change; p≤0.01) at (a) 14 and (b) 28 DAA. The rest of the details are as given in figure 4. Figure S3. The similarity search in Genevestigator, using the differentially expressed transcripts (IITR26 vs. WL711; ≥2 log2 fold change; p≤0.01) at 14 DAA revealed perturbations (top 5) in which spikelet samples of wheat genotypes, with stress resistant (CS-7EL) and susceptible (CS) backgrounds (GEO accession GSE21386), have been compared. Table S1. Gene-specific primers used for qRT-PCR. Table S2. Table S1 Differentially regulated probe sets with ≥2 log2 fold change expression difference at p≤0.01, between IITR26 vs. WL711, and their putative gene function during 14 and 28 DAA. Table S3. Annotation of the probe sets mentioned in Figure 5. (ZIP) [file pone.0111718.s001.zip › Table S1.docx]

**Table S1** Gene-specific primers used for qRT-PCR

Name of Gene Primers (5’– 3’) Amplicon size (in bp)

Ta.37139.1. Forward: ACTAACCGAGCCACGACTTCCGGC 165

Reverse : CCTATACTCGTGCATGACCCAGTTGGTC

Ta.12657.1 Forward: CGTGCAGTACAATAAGGACGACGGCC 156

Reverse : TGCAGATTCAGGTGCTGCGTCGGGA

Ta.28347.1 Forward: GCGAGCACGAGGATGAGGAGGAGAA 136

Reverse : CGTACAGAACTAAGGAACACATGACGGTT

Ta.29726.2 Forward: GGGGTTCGTCGGCTTCTTCGTCAAG 149

Reverse : CCACAGATGAGACAAAGGACGCACAC

Ta.8258.2 Forward: ACTGCTAGACCGAGTATGGCTCAGCTATA 145

Reverse: ACACATAACAGGGAACATGCGTGCGTG

|  |
| --- |
