# Supplementary material for: Comparative Transcriptional Profiling of Two Wheat Genotypes, with Contrasting Levels of Minerals in Grains, Shows Expression Differences during Grain Filling
Source: PLoS One. 2014 Nov 3;9(11):e111718. doi: 10.1371/journal.pone.0111718 (PMC4218811; doi:10.1371/journal.pone.0111718)
Supplement: File S1 — Contains the following files: Figure S1. Hierarchically clustered heat-map for 580 differentially expressed transcripts (IITR26 vs. WL711; ≥2 log2 fold change; p≤0.01) at 14 and 28 DAA. Figure S2. A heat map of differentially expressed transcripts (IITR26 vs. WL711; ≥2 log2 fold change; p≤0.01) at (a) 14 and (b) 28 DAA. The rest of the details are as given in figure 4. Figure S3. The similarity search in Genevestigator, using the differentially expressed transcripts (IITR26 vs. WL711; ≥2 log2 fold change; p≤0.01) at 14 DAA revealed perturbations (top 5) in which spikelet samples of wheat genotypes, with stress resistant (CS-7EL) and susceptible (CS) backgrounds (GEO accession GSE21386), have been compared. Table S1. Gene-specific primers used for qRT-PCR. Table S2. Table S1 Differentially regulated probe sets with ≥2 log2 fold change expression difference at p≤0.01, between IITR26 vs. WL711, and their putative gene function during 14 and 28 DAA. Table S3. Annotation of the probe sets mentioned in Figure 5. (ZIP) [file pone.0111718.s001.zip › Table S3.docx]

**Table S3** Annotation of the probe sets mentioned in Figure 5

Ta.9561.3.S1_a_at: Peroxin

Ta.28543.1.A1_at: Protein phosphatase type 2c

Ta.3862.1.S1_at: Protein phosphatase

Ta.24550.2.S1_s_at: Transition metal ion binding

Ta.5280.3.S1_at: Manganese ion binding

Ta.28347.1.S1_s_at: Metallothionein

Ta.10548.1.S1_at: Glycolate oxidase

Ta.2895.1.S1_x_at: Plasma membrane intrinsic protein

Ta.3209.2.S1_x_at: Stress-responsive-Retrotrnaposon

Ta.9898.1.A1_at: Vacuolar-sorting receptor precursor

Ta.1763.2.S1_at: Ras-related protein

Ta.14243.1.A1_at: Flavanone 3-hydroxylase

Ta.8881.1.S1_at: Dihydroflavonol-4-reductase

Ta.8472.1.S1_a_at: Ankyrin repeat protein

Ta.23465.1.S1_at: Ankyrin repeat family protein

Ta.488.3.S1_a_at: Peroxidase precursor

Ta.23366.2.S1_at: Peroxidase precursor

Ta.26351.1.A1_at: Glucan synthase-like

Ta.21557.1.A1_at: Senescence/dehydration associated protein

Ta.12657.1.S1_at: Lea protein12

Ta.5873.2.S1_at: Endochitinase

Ta.23888.1.S1_at: Basic endochitinase

Ta.1208.1.S1_at: Chitinase family protein

Ta.1200.1.S1_x_at: Xylanase inhibitor

Ta.14239.1.S1_at: Calcium/calmodulin depedent protein kinases

Ta.10310.3.S1_at: ABA related

Ta.9226.1.S1_at: Wheatwin

Ta.23141.1.S1_at: Puroindoline-A

Ta.28209.2.S1_x_at: BURP domain-containing protein

Ta.23322.1.S1_s_at: Thaumatin-like protein

Ta.26119.1.A1_at: 3-isopropylmalate dehydrogenase

Ta.5920.1.S1_at: ABA related-HVA22

Ta.5627.1.S1_x_at: VAMP protein

Ta.5578.1.S1_x_at: Disulfide oxidoreductase

Ta.5578.1.S1_x_at: Malic enzyme

Ta.5600.1.S1_s_at: Salt tolerance protein

Ta.368.1.S1_x_at: Calreticulin1

Ta.6822.1.S1_at: Cinnamoyl-CoA reductase

Taaffx.35126.1.A1_at: Bifunctional dihydroflavonol 4-reductase flavanone 4-reductase

Ta.1944.1.S1_at: Superoxide dismutase

Ta.10054.1.S1_at: ABA response element binding factor

Ta.5757.1.S1_at: stem-specific protein TSJT1
